# Hydrothermal Conversion of Wastewater Treatment Sands into Dual-Phase FAU/LTA Zeolite: Structural Insights and Performance in Methylene Blue Adsorption

**DOI:** 10.3390/molecules31030437

**Published:** 2026-01-27

**Authors:** Diana Guaya, María José Jara, José Luis Cortina

**Affiliations:** 1 Department of Chemistry, Universidad Técnica Particular de Loja, Loja 110107, Ecuador; 2 Escuela de Ingeniería Química, Universidad Técnica Particular de Loja, Loja 110107, Ecuador; 3 Department of Chemical Engineering, Polytechnic University of Catalonia–BarcelonaTech (UPC), 08019 Barcelona, Spain; jose.luis.cortina@upc.edu; 4 Barcelona Research Center in Multiscale Science and Engineering (CCEM), Universitat Politècnica de Catalunya-BarcelonaTech (UPC), Av. Eduard Maristany, 16, 08019 Barcelona, Spain

**Keywords:** residual sands, zeolite, FAU, LTA, methylene blue adsorption

## Abstract

This study presents a sustainable valorization strategy for wastewater treatment plant (WWTP) residual sands through their hydrothermal conversion into a dual-phase FAU/LTA zeolite and evaluates its adsorption performance toward methylene blue (MB) as a model cationic contaminant. The synthesized material (ZEO-RS) exhibited a low Si/Al ratio (~1.7), well-developed FAU supercages with minor LTA domains, and high structural integrity, as confirmed by XRD, FTIR, XRF, SEM and PZC analyses. ZEO-RS demonstrated rapid adsorption kinetics, reaching approximately 92% of equilibrium uptake within 30 min and following a pseudo-second-order kinetic model (k_2_= 2.73 g·mg^−1^·h^−1^). Equilibrium data were best described by the Langmuir isotherm, yielding a maximum adsorption capacity of 34.2 mg·g^−1^ at 20 °C, with favorable separation factors (0 < r_L_ < 1), while Freundlich fitting indicated moderate surface heterogeneity. Thermodynamic analysis revealed that MB adsorption is spontaneous (ΔG° = −11.98 to −12.56 kJ·mol^−1^), mildly endothermic (ΔH° = +5.26 kJ·mol^−1^), and entropy-driven (ΔS° = +0.059 kJ·mol^−1^·K^−1^). FTIR evidence, combined with pH-dependent behavior, indicates that adsorption proceeds via synergistic electrostatic attraction, pore confinement within FAU domains, and partial ion-exchange interactions. Desorption efficiencies conducted under mild acidic, neutral, and alkaline conditions resulted in low MB release (1–8%), indicating strong dye retention and high framework stability. Overall, the results demonstrate that WWTP residual sands are an effective and scalable low-cost precursor for producing zeolitic adsorbents, supporting their potential application in sustainable water purification and circular-economy-based wastewater treatment strategies.

## 1. Introduction

The rapid expansion of urbanization and industrial activities has intensified the demand for efficient wastewater treatment systems to safeguard public health and prevent environmental degradation [1,2,3,4]. Wastewater treatment plants (WWTPs) play a crucial role in this process; however, they generate large volumes of solid by-products, mainly sewage sludge and residual sands [5,6]. While sludge has been partially valorized through energy recovery, composting, or incorporation into construction materials, residual sands remain largely underutilized [7,8,9]. Their uncontrolled disposal in landfills or aquatic environments can lead to heavy metal leaching, particulate matter release, and secondary pollution, resulting in soil contamination and ecological disturbance. Nonetheless, given their high silica and alumina contents, these sands constitute a promising secondary raw material that can be transformed into high-value products such as construction aggregates, filtration media, catalytic supports, or zeolitic adsorbents, thereby supporting a circular economy approach to waste management [6,10].

Residual sands, often regarded as inert waste, are rich in SiO_2_ and Al_2_O_3_, making them excellent feedstocks for zeolite synthesis [11,12]. Traditionally, zeolites have been synthesized using high-purity chemical reagents (e.g., sodium silicate and aluminum hydroxide), which ensure controlled crystallinity but increase production costs and environmental impact [13,14]. As an alternative, natural and industrial by-products such as kaolin, fly ash, volcanic tuffs, and mining residues have been explored as low-cost sources of silicon and aluminum [15,16,17,18,19]. Although these raw materials reduce chemical consumption and waste generation, they often exhibit heterogeneous composition, lower purity, and variable Si/Al ratios, which may affect crystallization behavior and structural uniformity [20,21]. In this context, WWTP residual sands emerge as an untapped, compositionally stable, and locally available source for zeolite production. Their valorization not only mitigates the environmental burden associated with disposal but also enables the development of sustainable zeolitic materials with functional properties suitable for environmental remediation applications such as adsorption, ion exchange, and catalysis [22,23,24,25].

Among the various environmental applications of zeolitic materials, their use as adsorbents for organic dyes has been extensively documented due to their structural versatility, abundance of active sites, and excellent chemical and thermal stability [26,27,28,29,30]. Dyes, particularly those released by the textile, paper, and leather industries, represent one of the most persistent and hazardous classes of pollutants, characterized by high chromaticity, molecular complexity, and resistance to biodegradation [31,32,33]. Conventional treatment methods, such as coagulation–flocculation, chemical oxidation, and biological degradation, are often insufficient for complete dye removal, as they generate secondary sludge or exhibit limited selectivity toward recalcitrant molecules [34,35,36]. In this context, zeolite-based adsorption emerges as a promising alternative owing to its efficiency, regenerability, and operational simplicity [29,37]. However, zeolites synthesized from waste-derived precursors, while economically and environmentally advantageous, may display variations in purity, Si/Al ratio, and surface properties that influence adsorption capacity and reproducibility. According, the development of tailored zeolites with controlled composition and morphology from unconventional precursors, such as wastewater treatment residual sands, represents a critical step toward achieving high-efficiency, selective adsorption processes capable of addressing the current limitations of conventional dye removal technologies [38,39,40,41].

Methylene blue (MB) is a cationic thiazine dye widely used in the textile, paper, and pharmaceutical industries and is recognized for its high water solubility, intense chromaticity, and chemical stability [42,43,44]. These properties allow MB to persist in treated effluents [31,45], and when discharged into aquatic ecosystems, it can impede light penetration, reduce photosynthetic activity, and exert toxic, mutagenic, and neurotoxic effects on aquatic organisms and humans [46,47]. Its recalcitrant nature and strong interactions with organic and inorganic matter make MB an ideal model contaminant for evaluating the performance of emerging adsorbent materials [45,48,49]. Zeolites have been widely explored for MB removal owing to their negatively charged aluminosilicate frameworks, high cation-exchange capacity, and well-defined microporous structure, which promote electrostatic attraction and molecular sieving effects [50,51,52]. Various zeolitic frameworks, including clinoptilolite, mordenite, LTA, and FAU-type zeolites, have demonstrated significant adsorption capacities toward cationic dyes [53,54,55,56].

Among these materials, FAU-type zeolites (e.g., FAU-X and FAU-Y) are particularly attractive due to their three-dimensional pore system composed of large supercages (≈13 Å) interconnected by 12-membered oxygen rings, which enable the diffusion of relatively large dye molecules such as MB [57,58,59]. Their low Si/Al ratio further enhances the density of negative charge sites, promoting strong electrostatic interactions with cationic species [60,61]. Likewise, LTA-type zeolites, characterized by cubic morphology and 8-membered ring apertures (≈4.1 Å), exhibit high selectivity for small cations and dyes under optimized conditions [52,62]. Although their smaller pore openings compared to FAU-type structures limit the diffusion of bulky molecules, their surface charge, hydrophilicity, and tunable crystallite size can be engineered to improve adsorption performance [63,64,65]. The coexistence of FAU and LTA phases within a single material therefore offers a synergistic combination of large-pore and small-pore adsorption domains, facilitating the removal of cationic dyes with different molecular dimensions [41,66,67,68].

The novelty of this work lies in the conversion of wastewater treatment plant (WWTP) residual sands, an abundant yet underexplored waste stream, into dual-phase zeolitic materials (FAU–LTA) with tunable textural and adsorption properties. In contrast to conventional syntheses that rely on high-purity chemical reagents such as sodium silicate or aluminum hydroxide, or on industrial by-products such as fly ash and metakaolin, this study demonstrates a cost-effective and environmentally benign alternative that uses WWTP sands as the sole source of silicon and aluminum. These residual sands provide a stable and compositionally balanced precursor, eliminating the need for additional reagents and substantially reducing both material and energy consumption during synthesis. This approach represents a novel route that integrates waste valorization, green chemistry, and functional material design within a single framework [69,70]. By transforming an often-neglected waste into a high-performance zeolitic adsorbent, this strategy not only reduces the environmental impacts associated with disposal but also expands the portfolio of viable secondary raw materials for zeolite production [41,52]. Moreover, the successful synthesis of FAU–LTA structures from WWTP sands highlights their potential as sustainable substitutes for conventional, high-cost feedstocks, paving the way toward scalable and resource-efficient production of advanced zeolitic materials.

Therefore, this study aims to develop and characterize zeolitic materials derived from wastewater treatment plant (WWTP) residual sands through an optimized hydrothermal synthesis route. Specifically, the objectives are to: (i) evaluate the chemical and structural transformation of the sands into dual-phase zeolites (FAU–LTA); (ii) determine their physicochemical properties through XRF, XRD, FTIR, and PZC analyses; and (iii) assess their adsorption performance toward methylene blue (MB) as a model cationic pollutant. By integrating material design with environmental functionality, this work proposes an innovative valorization pathway for an underutilized waste stream while advancing the sustainable synthesis of zeolites from unconventional precursors. The outcomes of this research are expected to strengthen the scientific understanding of waste-derived zeolitic systems and to support the long-term development of scalable, low-cost adsorbents for water purification and circular resource management. Methylene blue was selected as a model contaminant because it is a well-established probe molecule for evaluating the adsorption performance of low-silica zeolites toward cationic species. Given the intrinsic negative framework charge and high cation-exchange capacity of FAU- and LTA-type zeolites, the evaluation of MB adsorption provides a representative and mechanistically relevant assessment of the material’s adsorption behavior toward cationic pollutants.

## 2. Results and Discussion

### 2.1. Physicochemical Characterization of Synthesized Zeolite

#### 2.1.1. Chemical Composition

The pretreated sands were primarily composed of SiO_2_ (68.2%) and Al_2_O_3_ (13.4%), along with minor fractions of CaO, Fe_2_O_3_, and K_2_O, as summarized in Table 1. After hydrothermal synthesis, the SiO_2_ content decreased to 41.4%, while Al_2_O_3_ increased to 24.1%, resulting in a SiO_2_/Al_2_O_3_ molar ratio of 1.7. This compositional shift reflects the partial dissolution of silicate phases and the incorporation of additional aluminum from sodium aluminate, promoting the crystallization of a low-silica zeolite framework. Zeolitic materials with SiO_2_/Al_2_O_3_ molar ratios in the range of 1.5–2.0 correspond to low-silica frameworks such as Na-A (LTA), Na-X (FAU), and P1-type zeolites, which exhibit a high density of framework negative charges and strong cation-exchange capacity [71]. Similar compositions have been widely reported for materials synthesized from aluminosilicate-rich residues, including coal fly ash, kaolin, and volcanic tuffs, where the aluminum enrichment promotes the formation of zeolitic structures with enhanced hydrophilicity and electrostatic affinity toward cationic pollutants [17,72,73]. For instance, Pérez-Botella et al. (2022) demonstrated that zeolites with Si/Al ratios below 2 exhibit enhanced ion-exchange performance due to the larger proportion of AlO_4_^−^ units [74], while Kordala (2024) and Li et al. (2023) reported the preferential crystallization of FAU- and LTA-type zeolites under comparable compositional conditions from waste-derived aluminosilicates [61,71]. Similarly, studies on zeolite synthesis from industrial wastes have shown that such Si/Al ratios favor the formation of highly reactive, Al-rich frameworks [38]. Therefore, the SiO_2_/Al_2_O_3_ ratio of 1.7 obtained in this study aligns well with the compositional domain of low-silica zeolites, confirming that the synthesis condition promoted aluminum incorporation and the crystallization of a cation-selective aluminosilicate network. Such low-silica compositions typically yield structures with stronger electrostatic interactions, greater hydrophilicity, and a higher density of accessible active sites than high-silica zeolites, which are more hydrophobic and exhibit lower cation affinity [74]. These results confirm that the applied synthesis conditions effectively facilitated the formation of a zeolite framework suitable for adsorption and ion-exchange applications [61].

#### 2.1.2. XRD Analysis

**Figure 1:**
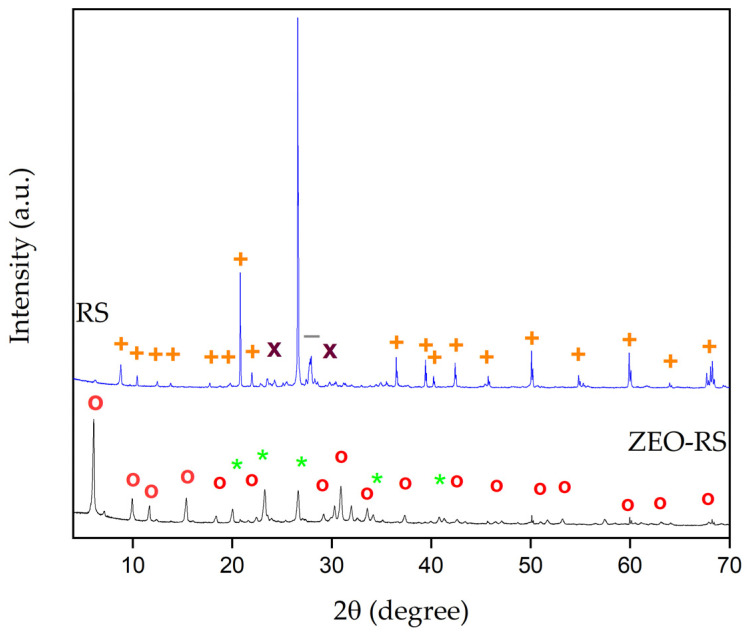
XRD patterns of residual sand (RS) and synthesized zeolite (ZEO-RS). Quartz (**+**), Anorthite (**X**), Muscovite (**−**), Faujasite (FAU, **o**) and Linde Type-A (LTA, *) zeolites.

The X-ray diffraction (XRD) patterns of the residual sand (RS) and the synthesized zeolite (ZEO-RS) are presented in Figure 1. The diffraction peaks of RS were indexed by comparison with reference database codes from the Crystallography Open Database (COD) [75], confirming quartz (COD 1501650) as the dominant crystalline phase. Quartz was identified as a monoclinic structure (space group P 1 21/c 1) with unit cell parameters a = 1.86 Å, b = 14.09 Å, c = 13.47 Å. Characteristic reflections were observed at 2θ ≈ 8.8°, 9.7°, 10.4°, 12.5°, 13.8°, 17.7°, 18.8°, 20.8°, 22.8°, 25.5°, 37.7°, 39.4°, 40.2°, 42.4°, 45.4°, 49.2°, 54.8°, 60.7°, 64.7°, and 68.3°. Additional reflections correspond to muscovite (COD 11000014), a trigonal aluminosilicate (P 31 1 2, a = b = 5.20 Å, c = 29.97 Å), with characteristic reflections at 2θ ≈ 24.1° and 28.06°, and anorthite (COD 9016764), a triclinic feldspar-type phase (P–1, a = 8.18 Å, b = 8.77 Å, c = 9.44 Å), with characteristic reflection at 2θ ≈ 27.9°. These results confirm the heterogeneous aluminosilicate nature of the precursor material, with the residual sand consisting primarily of well-crystallized silicate and aluminosilicate minerals, in agreement with its high SiO_2_ and Al_2_O_3_ contents determined by XRF. Such mineral phases are typical of sand-based residues from wastewater treatment plants and exhibit high crystallinity and structural stability, which limit their direct reactivity under alkaline conditions.

After hydrothermal treatment, the ZEO-RS diffraction pattern showed a complete transformation of the crystalline profile, with the disappearance of quartz reflections and the emergence of new, well-defined peaks characteristic of zeolitic structures. The appearance of these peaks at low 2θ angles (6–35°) indicates the formation of a highly ordered aluminosilicate framework, confirming the successful conversion of the inert precursor into a crystalline zeolite. The observed crystallographic features suggest the coexistence of multiple aluminosilicate domains, reflecting a competitive crystallization process influenced by the SiO_2_/Al_2_O_3_ ratio (≈1.7) and synthesis alkalinity. Comparison with reference patterns from the Database of Zeolite Structures of the International Zeolite Association (IZA) revealed that the materials consist predominantly of Faujasite (FAU-X) with a minor contribution from Linde Type-A (LTA) [76], both exhibiting cubic symmetry (Fd–3 m and Pm–3 m, respectively; a = b = c = 24.36 Å). FAU was identified by characteristic diffraction peaks at 2θ ≈ 6.1°, 10.1°, 12.3°, 16°, 17.5°, 23.3°, 29.2°, 32.5°, 34.1°, 42.6°, 47.1°, 51.1°, 54.6°, 60.5°, 63.2°, and 67.9°. In contrast, weak reflections corresponding to LTA were observed at 2θ ≈ 21.3°, 23.5°, 26.6°, 33.2°, and 40.8°, indicating the coexistence of both zeolitic frameworks. Quantitative phase analysis performed using HighScore Plus 5.3. indicated that the zeolitic product consists of approximately 98 wt% FAU and 2 wt% LTA, confirming the predominance of the faujasite framework. This phase composition is consistent with previous studies showing that hydrothermal synthesis under moderate alkalinity and low Si/Al ratios (≈1.5–2.0) favors FAU crystallization as the thermodynamically dominant phase, with LTA forming as a minor metastable phase [77,78]. Dual-phase FAU/LTA systems are commonly reported for zeolites derived from natural or residual silicates, where competitive crystallization and dissolution–reprecipitation mechanism governs phase evolution [70,79,80]. These XRD results confirm that the hydrothermal treatment of WWTP sands leads to a well-crystallized zeolite framework, representing a complete structural reorganization from quartz- and feldspar-based phases to FAU/LTA zeolitic lattices. The high crystallinity and absence of unreacted phases highlight the efficiency of the synthesis process and indicate excellent structural ordering [81].

#### 2.1.3. FTIR Analysis

**Figure 2:**
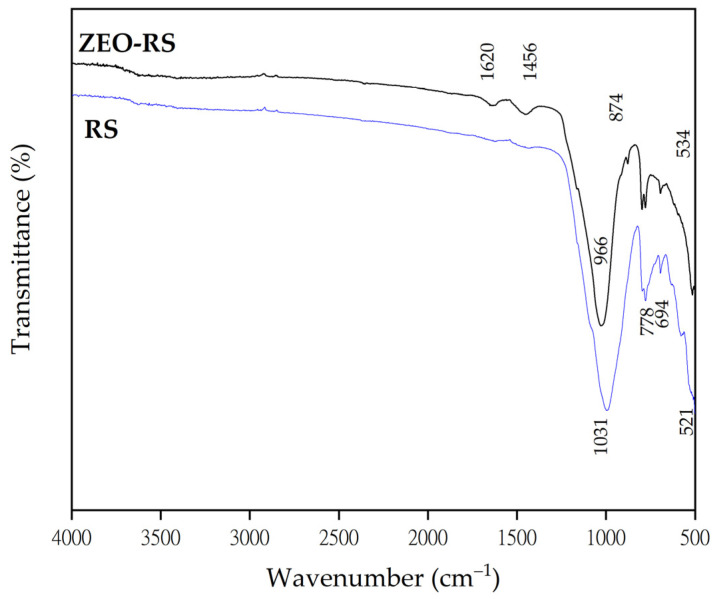
FTIR spectra of pretreated residual sand (RS) and synthesized zeolite (ZEO-RS).

The FTIR spectra of the residual sand (RS) and the synthesized zeolite (ZEO-RS) are compared in Figure 2. The RS spectrum exhibits a dominant asymmetric stretching band at approximately 1031 cm^−1^, typically attributed to Si–O–Si and Si–O–Al linkages in crystalline silicates such as quartz and feldspars, and characteristics of aluminosilicate networks present raw sand matrices [82,83]. A secondary band observed at around 521 cm^−1^ further confirms the presence of Si–O–Al bending vibrations, consistent with previously reported FTIR characterizations of aluminosilicate minerals [84].

In contrast, the FTIR spectrum of the synthesized material (ZEO-RS) shows significant modifications relative to the precursor, notably the appearance and shifting of absorption bands at approximately 966 cm^−1^, ~874 cm^−1^ and ~534 cm^−1^, together with weaker bands at around 1456 cm^−1^ and ~1620 cm^−1^. The shift in the main asymmetric Si–O–T stretching band toward lower wavenumbers (~966 cm^−1^) is indicative of increased aluminum incorporation and framework rearrangement, in agreement with systematic studies correlating the Si/Al ratio with this vibrational frequency in zeolitic materials [20,85].

The band near ~874 cm^−1^ and the bending vibration at approximately 534 cm^−1^ correspond to T–O (T = Si or Al) vibrations in tetrahedral units and to ring deformations typical of zeolitic frameworks rather than unmodified silicate minerals. For LTA-type zeolites, bands in the range of 500–800 cm^−1^ are characteristic of symmetric stretching vibrations of four-membered rings, as well as vibrations associated with double six-membered ring (D6R) units [85,86]. Similarly, in LTA and FAU-X zeolites, characteristic bands are commonly reported near ~1007 cm^−1^ and ~547 cm^−1^. The broad absorption region between approximately 3000 and 3500 cm^−1^ indicates the presence of surface hydroxyl groups and adsorbed water, which is typical of hydrated zeolites with open framework structures [20,85].

The weak bands at around 1620 cm^−1^ and 1456 cm^−1^ are attributed to the H–O–H bending modes of confined water molecules and to bending vibrations of residual hydroxyl groups, respectively [85,87]. Their presence further supports the formation of a zeolitic framework and confirms the successful transformation of the precursor material into a hydrated aluminosilicate structure.

#### 2.1.4. SEM Analysis

**Figure 3:**
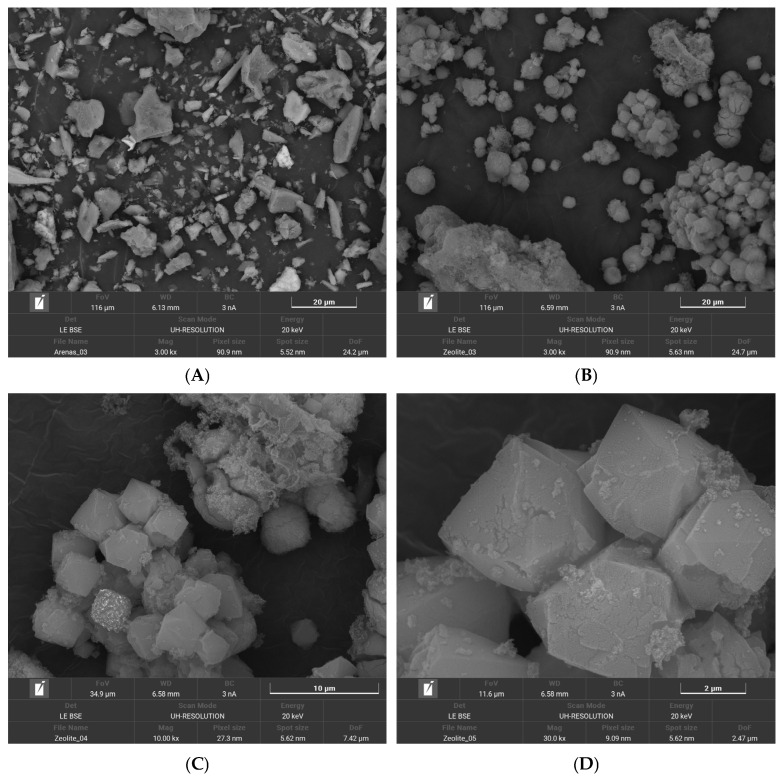
SEM micrographs of (**A**) pretreated residual sand (RS) and (**B**) synthesized zeolite (ZEO-RS), scale bar = 20 µm, magnification: ×3000; (**C**,**D**) ZEO-RS at scale bars of 10 µm (×10,000) and 2 µm (×30,000).

The morphological features of the residual sand (RS) and the synthesized zeolite (ZEO-RS) are shown in Figure 3. The SEM image of the raw material (RS) reveals heterogeneous, irregularly shaped particles with angular edges and compact, nonporous surfaces (Figure 3A). This morphology is characteristic of inert silicate mineral aggregates composed mainly of quartz, feldspar, and aluminosilicate fragments [88], consistent with residues commonly found in wastewater treatment plants (WWTPs). The dense texture indicates a low specific surface area and limited number of accessible active sites, a behavior similarly reported for untreated aluminosilicate residues such as fly ash prior to alkaline activation [89].

After hydrothermal synthesis, a marked morphological transformation is observed in the ZEO-RS sample (Figure 3B,C,D). The SEM images reveal aggregates of well-defined particles corresponding to zeolitic frameworks of the FAU and LTA types, in agreement with the XRD results [20,69,80]. The faujasite-type crystals appear as large, polyhedral and faceted aggregates with relatively smooth surfaces and particle sizes ranging from 1 to 3 µm, often forming intergrown clusters [82]. This morphology is characteristic of faujasite-type zeolites crystallized under moderate alkalinity and Si/Al ratios near 1.5–2.0, as previously reported for faujasites synthesized from aluminosilicate wastes, which exhibit similar octahedral or polyhedral shapes [15,69]. The presence of these well-faceted grains suggests a highly ordered crystallization process, consistent with the strong crystallinity observed by XRD and the low Si/Al ratio obtained from XRF analysis (≈1.7). In comparison, faujasite-Y zeolites (FAU-Y), typically synthesized at higher Si/Al ratios (2.5–3.0) and under more silica-rich or highly alkaline conditions, tend to exhibit smaller, less uniform crystallites with rougher surfaces and a greater tendency to form spherical or irregular aggregates [19]. Therefore, the predominance of large, faceted polyhedral particles in ZEO-RS is indicative of a low-silica faujasite-X structure (FAU-X), in agreement with the compositional and spectroscopic evidence.

The LTA crystals are distinguished by their well-defined cubic morphology with sharp edges and relatively uniform particle sizes ranging from approximately 0.5 to 2 µm. These cubic particles frequently appear densely packed or partially intergrown with faujasitic domains, suggesting simultaneous nucleation and competitive growth of both zeolitic structures during the hydrothermal synthesis process [78,79,80]. This morphology is consistent with typical LTA crystal structures, which are commonly obtained under high-alkalinity and low Si/Al ratio conditions (≈ 1.0–1.5) [78,90].

The Si/Al ratio determined for ZEO-RS (≈ 1.7) falls close to this compositional domain, supporting the coexistence of LTA and FAU phases as low-silica zeolitic domains. The agreement between chemical composition and crystal morphology reinforces the interpretation that both structures are present in the synthesized material. Overall, SEM observations confirm that hydrothermal treatment effectively transformed the compact, inert sand particles into well-crystallized zeolitic materials with distinct morphological domains reflecting the FAU/LTA dual-phase structure. The coexistence of both cubic LTA and polyhedral FAU-X crystals supports a competitive crystallization pathway typical of low-silica zeolites derived from natural or waste-based aluminosilicates under alkaline hydrothermal conditions [69,70,78]. Similar dual-phase morphologies have been reported for zeolites synthesized from a variety of raw materials, where FAU is often the dominant phase and LTA appears as a secondary phase [78]. In some cases, the initial nucleation of LTA followed by intercrystalline transformation into FAU has also been documented [80].

### 2.2. Adsorption Performance Evaluation

#### 2.2.1. Effect of pH on Adsorption Efficiency

**Figure 4:**
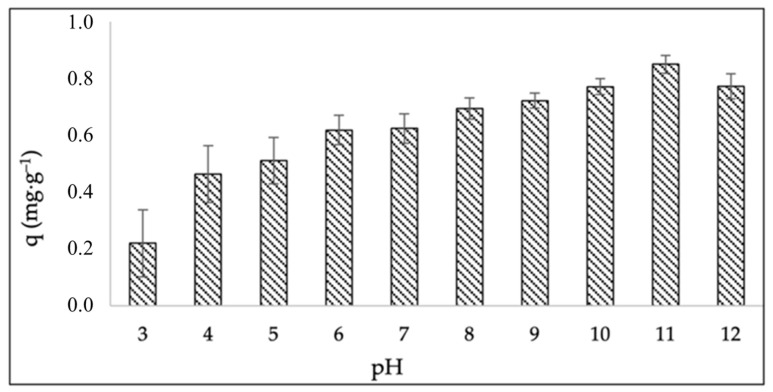
Effect of pH on methylene blue (MB) adsorption efficiency on ZEO-RS.

The adsorption of methylene blue (MB), a cationic thiazine dye, onto the synthesized zeolite (ZEO-RS) was strongly influenced by the pH, as shown in Figure 4. The point of zero charge (PZC) of ZEO-RS was determined to be 10.6 ± 0.2, indicating that the zeolite surface is positively charged at pH values below 10.6 and becomes negatively charged at pH values above this threshold. This shift in surface charge governs the electrostatic interactions between MB^+^ ions and the adsorbent’s active sites.

Comparable PZC values have been reported for low-silica, aluminum-rich zeolites such as FAU-X and LTA types, typically ranging between 9.5 and 11.0 [91,92], reflecting their strong basic character and high density of framework aluminum [93]. Therefore, the PZC obtained for ZEO-RS confirms its low-silica nature and the predominance of basic surface sites. This characteristic enhances electrostatic attraction between the negatively charged zeolite surface (pH > 10.6) and cationic MB species, thereby improving adsorption efficiency under alkaline conditions [52]. The pH-dependent adsorption study revealed a progressive increase in MB uptake with increasing pH, reaching maximum adsorption at pH 11.

At acidic to near-neutral pH values (pH 3–7), the adsorption capacity was relatively low because the surface of ZEO-RS remained positively charged, resulting in electrostatic repulsion between protonated zeolitic sites and MB^+^ molecules. In addition, under acidic conditions, H^+^ ions compete with MB^+^ species for available adsorption sites, further inhibiting dye uptake. Similar trends have been reported for cationic dye adsorption onto zeolitic and aluminosilicate materials with high PZC values [52]. These results confirm that electrostatic interactions play a major role in the adsorption of MB onto ZEO-RS, primarily governed by the protonation–deprotonation equilibrium of surface functional groups. These results confirm that electrostatic interactions play a major role in the adsorption of MB onto ZEO-RS, primarily governed by the protonation–deprotonation equilibrium of surface hydroxyl groups [55]. However, the noticeable adsorption observed at acidic pH values (pH < PZC), where electrostatic repulsion between positively charged ZEO-RS surfaces and MB^+^ would be expected, indicates that additional mechanisms may be involved [52]. As the pH increased toward alkaline conditions (pH > 8), the adsorption efficiency increased significantly. In this region, deprotonation of surface hydroxyl groups (≡Si–OH and ≡Al–OH) generates negatively charged sites (≡Si–O^−^ and ≡Al–O^−^), enhancing electrostatic attraction with MB^+^ ions. Maximum removal occurred near pH 10–11, where the surface charge of ZEO-RS is predominantly negative, facilitating strong dye–surface interaction through Coulombic forces [52]. Similar pH-dependent behavior has been reported for various zeolitic structures targeting MB removal [55]. Overall, electrostatic attraction is the dominant mechanism at alkaline pH values (pH > PZC); however, the adsorption behavior across the entire pH range suggests the contribution of additional interaction pathways. This highlights the structural versatility and complex surface chemistry of the synthesized zeolite, enabling effective MB uptake under varying pH conditions [53,54]. Despite the higher adsorption efficiency observed at alkaline conditions, pH 7 was selected for subsequent kinetic and equilibrium experiments to better represent environmentally relevant conditions and ensure practical applicability in wastewater treatment scenarios.

#### 2.2.2. Adsorption Kinetics

**Figure 5:**
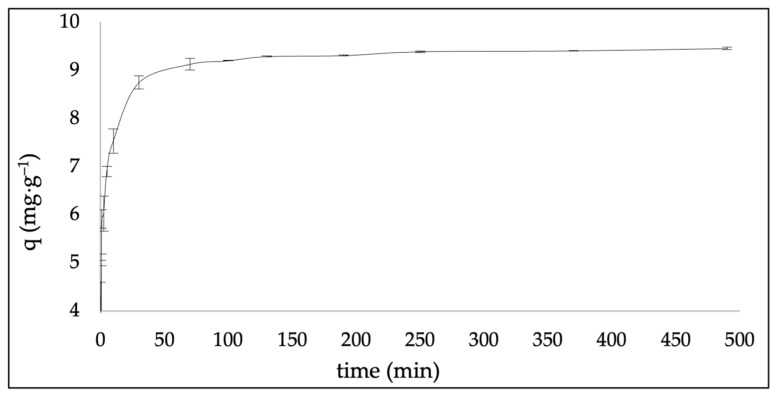
Adsorption kinetics of methylene blue (MB) onto ZEO-RS (data shown up to 8 h; adsorption equilibrium was experimentally confirmed for a total contact time of 24 h).

The adsorption kinetics of methylene blue (MB) onto the synthesized zeolite (ZEO-RS) are presented in Figure 5. Although the adsorption experiments were conducted for a total contact time of 24 h, the kinetic profile in Figure 5 is shown up to 8 h, as the adsorption capacity remained practically constant beyond this time and no significant variation was observed between 8 h and 24 h. The adsorption capacity (qₜ) increased rapidly during the first 30 min, reaching approximately 92% of the total uptake, and gradually approached equilibrium after about 120 min. This rapid initial uptake suggests that the early stage of adsorption is dominated by electrostatic attraction between the negatively charged zeolitic surface and cationic MB^+^ molecules. Comparable rapid adsorption stages have been previously reported for zeolites synthesized from industrial residues [52,94].

Beyond 120 min, the adsorption curve reached a plateau, indicating equilibrium, with a maximum capacity of approximately 9.8 mg·g^−1^. At this stage, most readily accessible active sites, including external exchange positions and micropore entrances, were occupied, and mass transfer resistance became the dominant limiting factor. As the number of available sites decreases, dye molecules require longer diffusion times to access deeper regions of the internal pore system. This transition from surface-controlled to diffusion-controlled kinetics is characteristic of low-silica zeolites, where a high density of exchangeable cations promotes rapid initial binding followed by a slower, diffusion-driven approach to equilibrium [51,95]. Similar equilibrium plateaus and diffusion-limited behavior have been widely reported for MB adsorption on zeolitic materials derived from industrial residues [52].

The kinetic parameters summarized in Table 2 indicate that MB adsorption onto ZEO-RS follows a pseudo-second-order kinetic model, as evidenced by the higher correlation coefficient (R^2^ = 0.99) compared to the pseudo-first-order model (R^2^ = 0.85). This suggests that the adsorption rate is governed predominantly by chemisorption process involving electron sharing or exchange between MB molecules and zeolitic active sites. This interpretation is consistent with the pH-dependent adsorption behavior (Figure 4), which indicates that non-electrostatic interactions contribute to MB uptake even under conditions where electrostatic repulsion may be expected.

The equilibrium adsorption capacity predicted by the pseudo-second-order model (Qₑ = 9.52 mg·g^−1^) closely matches experimental value, further confirming the suitability of this model. The relatively high-rate constant (K_2_ = 2.73 g·mg^−1^·h^−1^) reflects rapid interaction between MB molecules and reactive surface sites, consistent with adsorption mechanisms commonly reported for cationic dyes on aluminosilicate frameworks [50]. These mechanisms include ion-exchange with charge-compensating cations; Lewis acid–base interactions involving framework aluminum sites, hydrogen bonding with surface hydroxyl groups, and strong dye–surface associations within microporous regions [54,55].

Additional insights into the rate-controlling steps were obtained from the intraparticle-diffusion, film-diffusion and particle-diffusion models (Table 2). The multi-linear intraparticle-diffusion plot revealed three distinct regions: (i) an initial stage with a high diffusion constant (k_1_ = 9.37 g·mg^−1^·h^−1^), corresponding to rapid transport across the external surface and macropores; (ii) an intermediate stage with a lower diffusion constant (k_2_= 0.71 g·mg^−1^·h^−1^), associated with diffusion into microporous domains; and (iii) a final stage with the smallest diffusion constant (k_3_ =0.05 g·mg^−1^·h^−1^), corresponding to equilibrium and diffusion into the least accessible sites. This multistep diffusion behavior agrees with kinetic patterns reported for MB adsorption on NaX and LTA-type zeolites, where external-film transfer and intraparticle diffusion occur concurrently [52].

Film-diffusion and particle-diffusion models also confirmed the contribution of both external and internal mass-transfer resistances. The apparent film-diffusion coefficient (D_f_ = 2.5 × 10^7^ m^2^·h^−1^; R^2^ = 0.83) and the effective particle-diffusion coefficient (D_p_ = 2.5 × 10^10^ m^2^·h^−1^; R^2^ = 0.86) indicate that hydrodynamic boundary-layer transport and intraparticle diffusion jointly influence the overall kinetic process, consistent with previous reports on MB adsorption by zeolitic frameworks [52,96].

These kinetic results can be interpreted considering the molecular dimensions of MB and the pore architecture of the dual-phase FAU/LTA framework in ZEO-RS. MB has an approximate molecular size 1.43 × 0.61 × 0.40 nm (14.3 × 6.1 × 4.0 Å). FAU-type zeolites possess large supercages (~13 Å) accessible through 12-membered-ring windows (~7.4 Å), allowing diffusion of elongated MB molecules in favorable orientation [57,97]. In contrast, LTA-type zeolites contain α-cages (~11.4 Å) connected by narrower 8-membered-ring windows (~4.1 Å), which are comparable to or smaller than the smallest cross-section of MB, significantly restricting its internal diffusion [52,98]. Computational and experimental studies on MB adsorption onto zeolite LTA are largely limited to external surfaces and pore mouths [96,99].

In the dual-phase ZEO-RS system, MB molecules are therefore preferentially accommodated within FAU supercages, while LTA domains contribute mainly through adsorption at external surfaces. The combination of a rapid initial uptake dominated by surface interactions and a slower diffusion-controlled uptake inside the FAU domains explains both the observed kinetic behavior and multistage intraparticle diffusion profile. Overall, these results indicate that MB adsorption onto ZEO-RS proceeds through a combination of surface chemisorption and pore-diffusion mechanisms, confirming the effectiveness of the synthesized zeolite as a low-cost, aluminum-rich adsorbent for the removal of cationic contaminants aqueous systems [100,101].

#### 2.2.3. Isothermal and Thermodynamic Evaluation

The equilibrium adsorption data of methylene blue (MB) onto the synthesized zeolite (ZEO-RS) were fitted to both the Langmuir and Freundlich isotherm models (Table 3). The Freundlich model provides a stronger fit (R^2^ = 0.98) than the Langmuir model (R^2^ = 0.94–0.95), suggesting that adsorption occurs on a heterogeneous surface. The Freundlich parameter (n^−1^ = 0.80 and 0.87) further indicates moderate surface heterogeneity and adsorption behavior that deviates from ideal monolayer uptake.

The maximum adsorption capacity (q_m_) estimated from the Langmuir model ranged from 34.2 to 36.6 mg g^−1^ at 20–30 °C. The slight increase in q_m_ with temperature suggests an endothermic adsorption process, commonly associated with enhanced diffusion and electrostatically driven uptake. This trend is consistent with reports indicating that MB adsorption on low-silica aluminosilicate frameworks is largely governed by electrostatic attraction between negatively charged zeolitic sites and cationic dye molecules [54,96]. Previous studies have shown that MB adsorption capacity generally follows the order LTA < FAU-X < FAU-Y, which is primarily attributed to differences in pore aperture size and accessibility. LTA zeolites, with narrower 8-ring windows, can restrict the penetration of bulky dye molecules [52,55], whereas FAU-type zeolites provide larger 12-membered-ring windows and supercages that enhance molecular diffusion and uptake [100,101,102]. Accordingly, the q_m_ values obtained for ZEO-RS are consistent with those expected for FAU-dominant materials.

The Langmuir constants (k_L_ = 7.7–8.2 × 10^−3^ L·g^−1^) and Freundlich constants (k_F_ ≈ 0.31 mg·g^−1^) indicate moderate affinity, which supports efficient adsorption. Overall, these findings suggest that MB uptake is dominated physical interactions (electrostatic attraction and diffusion within microporous regions), with minor contributions from stronger interactions that may involve exchangeable framework sites.

Comparable adsorption capacities and mixed-model behavior have been reported for FAU-type zeolites, particularly when surface heterogeneity and multiphase compositions contribute to combined Langmuir–Freundlich characteristics [56,100]. Overall, the isotherm analysis confirms that ZEO-RS is an effective and sustainable adsorbent, exhibiting moderate adsorption capacity, good selectivity toward cationic dyes, and structural robustness associated with its FAU/LTA dual-phase framework. The favorability of MB adsorption on ZEO-RS was further assessed using the dimensionless Langmuir separation factor r_L_. The calculated r_L_ values, derived from the Langmuir constant and the corresponding initial MB concentrations, ranged between 0.91 and 0.99 for all investigated temperatures (Table 3). These values fall within the range 0 < r_L_ < 1, confirming that adsorption is favorable under the studied conditions. Because r_L_ values approach unity, they reflect a moderately favorable affinity rather than strongly irreversible uptake. Nevertheless, the consistently favorable r_L_ values across the evaluated temperatures support the applicability of the Langmuir model and confirm the practical suitability of the FAU/LTA zeolite for MB removal under realistic operating conditions.

The thermodynamic parameters obtained for MB adsorption onto ZEO-RS (Table 4) provide additional insight into the nature of the adsorbate–adsorbent interactions. The negative Gibbs free energy values (ΔG° = −11.98 to −12.56 kJ·mol^−1^) over 293–303 K confirm that adsorption is spontaneous and thermodynamically favorable [51]. The magnitude of ΔG°, which lies within the typical range associated with physisorption (0 to −20 kJ·mol^−1^), indicates that MB uptake is predominantly governed by weak and reversible interactions rather than strong chemisorption (often reported as >−80 kJ·mol^−1^). In combination with the kinetic and isotherm analyses, these results support adsorption dominated by physisorption, with possible secondary contributions from stronger site-specific interactions.

The positive enthalpy change (ΔH° = +5.26 kJ·mol^−1^) indicates an endothermic process, consistent with slightly improved adsorption at higher temperatures due to increased molecular mobility and enhanced diffusivity within zeolitic micropores. Such low positive enthalpy values are commonly associated with electrostatic and ion–dipole interactions and contrast with the higher ΔH° values (>80 kJ mol^−1^) typically attributed to chemisorption [51]. This further supports a physisorption-dominated process, coexisting with weak chemical interactions. Similar endothermic behavior has been reported for MB adsorption on FAU- and LTA-type zeolites, where temperature primarily enhances diffusion and interaction with hydrophilic pore surfaces [94,103].

The positive entropy change (ΔS° = 0.059 kJ·mol^−1^·K^−1^) reflects increased disorder at the solid–liquid interface during adsorption, as MB molecules transfer from the aqueous phase into the zeolitic environment [52,103]. Overall, the combination of spontaneous ΔG°, mildly endothermic ΔH°, and positive ΔS° indicates that MB adsorption onto ZEO-RS proceeds through an entropy-assisted, physisorption-dominated mechanism, supported by electrostatic attraction, pore confinement, and ion-dipole interactions [51,52,103]. These thermodynamic results are consistent with the isotherm and kinetic analyses and confirm the stability and effectiveness of ZEO-RS for removing cationic dyes from aqueous media.

#### 2.2.4. Regeneration and Reusability

**Figure 6:**
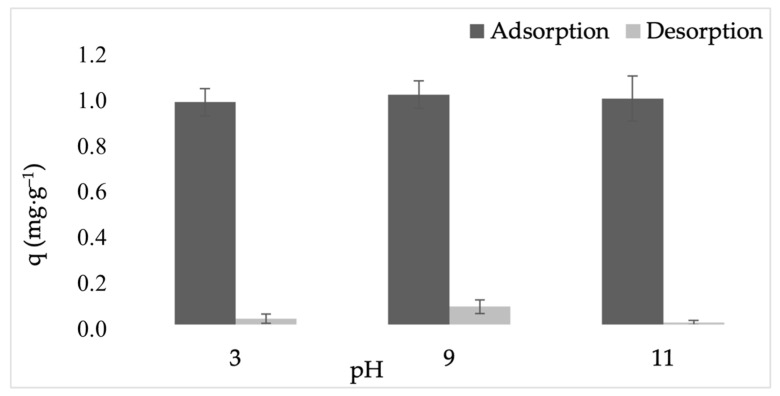
Adsorption–desorption behavior of MB on ZEO-RS at different pH values.

The desorption experiments were performed using aqueous acidic, neutral, and alkaline media deliberately selected to represent mild and environmentally benign conditions, thereby providing a conservative assessment of desorption behavior. Weak acidic and weak alkaline solutions were employed to evaluate pH-driven desorption mechanisms and to assess the stability of adsorbed methylene blue (MB) under different chemical environments, while avoiding aggressive treatments. The regeneration performance of ZEO-RS was evaluated through desorption assays conducted after dye adsorption at neutral conditions (pH 7). As shown in Figure 6, MB desorption remained very low across all tested pH values, with release percentages of ~3% at pH 3, ~8% at pH 9, and only ~1% at pH 11. These results indicate that MB is strongly retained within the FAU/LTA zeolitic microstructure and that the dye–surface interactions are not readily reversible under either acidic or alkaline conditions, consistent with the previous reports on MB adsorption by zeolites [52]. The use of mild aqueous solutions was motivated by the well-documented sensitivity of low-silica zeolites to strong acidic or highly alkaline environments, which can induce dealumination, framework degradation, or loss of crystallinity. A neutral aqueous solution was included as a reference condition to evaluate MB release in the absence of pH-driven desorption forces. Although previous studies have reported that saline electrolytes (e.g., NaCl), and organic solvents may enhance the desorption of cationic dyes, the use of such eluents may increase environmental burdens, operational complexity, and/or the risk of framework alteration. Therefore, these approaches were not investigated in the present work.

At pH 3, protonation of surface hydroxyl groups (≡Si–OH_2_^+^ and ≡Al–OH_2_^+^) generates a positively charged environment that suppresses dye release. Although electrostatic repulsion between the protonated surface and MB^+^ could theoretically promote desorption, the low release (~3%) suggests that a substantial fraction of MB remains confined within the micropores and/or associated with internal cavities and exchange sites. This behavior is characteristic of aluminum-rich, low-silica zeolites, where MB retention involves combined contributions from ion-exchange, van der Waals forces, and hydrogen bonding, which are not easily disrupted under acidic conditions [95,101].

At pH 9, the highest desorption (~8%) was observed. This moderate release can be attributed to partial deprotonation of ≡Si–OH and ≡Al–OH groups, which can weaken electrostatic interactions and facilitate the displacement of MB molecules located at external surfaces or near pore entrances. Nevertheless, MB accommodated within FAU supercages appears to remain strongly confined, consistent with previous reports describing limited reversibility of MB adsorption on FAU-X zeolites under mildly alkaline conditions [102].

At pH 11, desorption decreased sharply to ~1%, despite the highly negative surface charge expected under strongly alkaline conditions. This minimal release suggests that, after adsorption at neutral pH, MB penetrates into FAU supercages and/or occupies internal cation-exchange sites where steric confinement and site-specific interactions dominate. Under strong alkaline conditions, electrostatic stabilization between ≡Si–O^−^/≡Al–O^−^ sites and MB^+^ may further promote dye retention within the pore network, as reported for FAU- and LTA-type zeolites where steric restrictions and framework hydrophilicity limit reversibility [52,99,101].

Overall, the regeneration results indicate that MB adsorption on ZEO-RS is highly stable. Dye molecules are strongly retained through a combination of physical (pore confinement, ion–dipole interactions) and chemical contributions (ion-exchange) [99]. Although partial desorption was observed at pH 9, the overall low reversibility suggests that ZEO-RS is more suitable for single-use or limited-cycle adsorption applications, particularly where dye leaching must be minimized. This conclusion aligns with previous reports of limited MB desorption from FAU-type zeolites and supports the structural robustness and strong dye affinity of the synthesized material [52].

The low desorption across all tested pH conditions has important operational implications. In full-scale wastewater treatment systems, adsorbents with limited regeneration efficiency often function as quasi-single-use materials, especially when the adsorbed species exhibit strong affinity for internal zeolitic cavities, as is the case for MB within FAU/LTA frameworks. While this behavior ensures excellent stability and negligible dye leaching, it may increase long-term operational cost due to more frequent adsorbent replacement. However, low-cost zeolites produced from industrial residues, such as ZEO-RS, may remain economically attractive even when regeneration is limited, particularly in scenarios where contaminant immobilization, process simplicity, and high removal efficiency are prioritized [95]. Accordingly, ZEO-RS may be especially relevant for decentralized treatment units, polishing stages, or systems in which spent adsorbents can be safely valorized (e.g., incorporation into construction materials) or managed through controlled disposal strategies [104].

In this study, distilled water was selected as the desorbing medium due to its neutral free of interfering ions, and widely applied in standardized regeneration protocols for cationic dyes on zeolitic materials [52]. This approach provides a conservative evaluation of intrinsic reversibility by allowing desorption driven primarily by weak physical interactions, without contributions from competing ions or complexing agents. The negligible MB release observed confirms the strong affinity between MB molecules and internal FAU/LTA sites. Given the limited regeneration achieved under these conditions, future work should investigate more effective yet framework-compatible eluents, such as saline electrolytes, dilute acids or bases, or suitable organic solvents, that may enhance competitive ion exchange or weaken dye–surface interactions.

From a process-design perspective, it should be emphasized that the regeneration study was intentionally restricted to pH-controlled aqueous media to evaluate desorption under mild and environmentally compatible conditions. The effectiveness of alternative regeneration strategies involving high ionic strength, strong acids or bases, oxidizing agents, or thermal treatments was not assessed in this work and remains outside its scope.

Consequently, the low desorption efficiencies reported here should be interpreted not as a limitation of adsorption itself, but as an operational characteristic of ZEO-RS. This behavior indicates that the material is better suited for applications in which adsorbent replacement or final immobilization is preferred over repeated regeneration cycles, and where process simplicity, contaminant stability, and low-cost feedstock utilization outweigh the need for multi-cycle reuse.

#### 2.2.5. Literature Comparison of Methylene Blue Adsorption on Zeolitic Materials

To contextualize the methylene blue (MB) adsorption performance of the synthesized FAU/LTA zeolite, a comparative analysis was conducted using data reported in the literature. This comparison includes LTA-type, FAU-type (faujasite X) zeolites, and other related zeolitic materials synthesized from both waste-derived precursors and conventional raw materials.

Table 5 summarizes reported MB adsorption capacities together with BET surface areas and precursor origins; all values were strictly extracted from peer-reviewed studies. Given the diversity of experimental conditions across the literature, such as differences in initial MB concentration, solution pH, adsorbent dosage, and contact time, this comparison is intended as qualitative benchmarking rather than direct quantitative ranking. As shown in Table 5, the adsorption capacity obtained in the present study falls within the range reported for LTA- and FAU-type zeolites derived from various aluminosilicate sources, including industrial residues such as fly ash, mining tailings, and waste glass. Overall, these results indicate that the FAU/LTA material exhibits adsorption performance comparable to that reported for several single-phase zeolitic systems.

It is also noteworthy that adsorption performance is not governed solely by BET surface area or phase purity. Literature data indicate that framework topology, charge density, and accessibility of adsorption sites can significantly influence MB uptake. Reports specifically addressing MB adsorption on FAU-type zeolites, particularly faujasite X, remain relatively limited compared with other zeolite families, as highlighted in previous studies [105]. In this context, the performance achieved by the FAU/LTA zeolite further supports the relevance of waste-derived zeolitic materials synthesized through simple and sustainable routes.

Beyond adsorption capacity, the practical relevance of the proposed material is further supported by using wastewater treatment plant residual sands as a low-cost and readily available precursor, the simplicity of the synthesis process, and the absence of costly post-synthesis modification steps. These aspects collectively support the potential applicability of the FAU/LTA zeolite for environmentally relevant adsorption processes.

**Table 5:** Comparison of reported methylene blue adsorption capacities for zeolitic materials synthesized from waste-derived and conventional precursors.

Waste Precursor	Zeolitic Material (Phase)	BET Surface Area (m^2^·g^−1^)	MB Adsorption Capacity (mg·g^−1^)	Reference
Mining tailings	LTA zeolite	48	30	[41]
Mining tailings	LiOH-modified LTA zeolite (ZA-Li^+^)	52	43	
Pure reagents	NaX zeolite	375	24	[100]
Waste glass fiber	Zeolite-like mesoporous material (analcime-type)	166	132	[106]
Fly ash	Zeolite	132	0.7	[107]
Kaolin	Zeolite–X	7146	2	[105]
Phosphogypsum Flotation Tailings	Zeolite A	44.2	32	[108]
Rice hush ask (RHA)	Zeolite NaY	164.4	49	[95]
Clays	LTA (MZ)	-	77	[66]
	LTA (ABZ)	-	76	
	FAU Y (ANF)	-	79	
Kaolin	NaA	625	196	[109]
Waste coal post-combustion fly ash (Raw CFA)	Pure nanozeolite X (nFAZX)	770	345	[110]
	Commercial zeolite X (CZX)	763	250	
Huadian oil shale ash	NaX zeolite	367	50	[111]
Molybdenum tailings	Faujasite-Na zeolite	478	45	[112]
Wastewater treatment plan residual sands	ZEO–RS	51	34.2–36.6 (20–30 °C)	This study

From a practical and economic perspective, the FAU/LTA zeolite developed in this study presents several advantages over conventional adsorbents. The use of wastewater treatment plant residual sands as the aluminosilicate precursor significantly reduces raw material costs while simultaneously contributing to waste valorization and circular economy strategies. Unlike conventional zeolite syntheses based on high-purity reagents, the proposed approach relies on an abundant and locally available waste stream, which is particularly advantageous for large-scale implementation.

In addition, the hydrothermal synthesis route employed does not require organic structure-directing agents, complex purification steps, or post-synthesis functionalization, which are commonly associated with increased production costs. When combined with the competitive adsorption performance observed for methylene blue removal, these features suggest that the FAU/LTA zeolite offers a favorable balance between performance and cost. While a full techno-economic assessment is beyond the scope of this study, a qualitative comparison with conventional adsorbents highlights the potential feasibility of scaling up this material for practical wastewater treatment applications.

#### 2.2.6. Proposed Adsorption Mechanism

**Figure 7:**
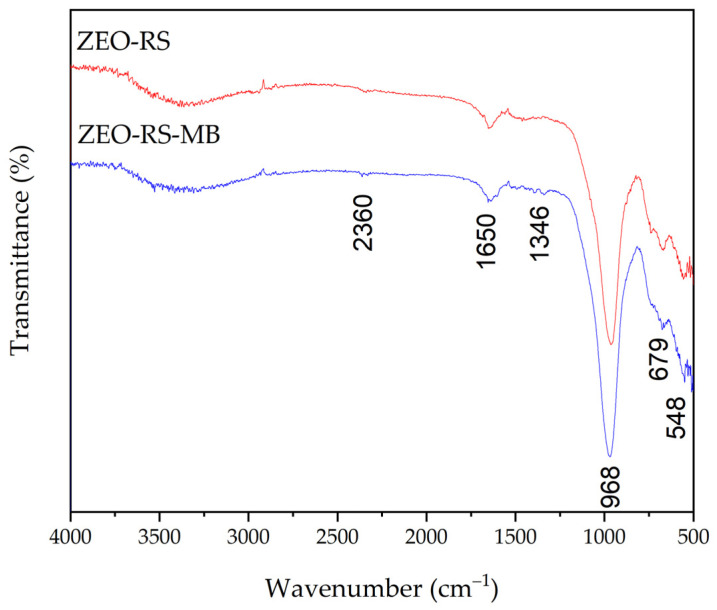
FTIR spectra of synthesized zeolite (ZEO-RS) and zeolite loaded with methylene blue (ZEO-RS-MB).

The adsorption mechanism of methylene blue (MB) on the dual-phase FAU/LTA zeolite was interpreted based exclusively on the experimental evidence obtained in this study. FTIR analysis comparing the pristine zeolite (ZEO-RS) and the MB-loaded material (ZEO-RS-MB) confirms that the zeolitic framework remains structurally intact after adsorption, as evidenced by the persistence of characteristic T–O–T framework vibrations (Figure 7) [52,103]. The pronounced decrease and broadening of the O–H stretching region (3000–3600 cm^−1^) after MB adsorption indicate the involvement of surface silanol and aluminol groups in the adsorption process. These spectral changes, together with the strong pH dependence of MB uptake, support the contribution of electrostatic attraction between negatively charged framework sites and cationic MB species [95,96]. The enhancement of the band at ~1620 cm^−1^ is attributed to changes in the environment of coordinated water molecules within the zeolite structure, which may occur upon MB incorporation into the internal cavities. This observation supports the contribution of pore confinement effects, particularly within FAU supercages, to the overall adsorption process [101,113]. In contrast, steric restrictions in LTA domains are consistent with limited MB penetration to external surfaces and pore mouths [66,100]. In addition, the appearance and intensification of bands in the 1450–1600 cm^−1^ region, corresponding to aromatic C=C and C–N vibrations, confirm the presence of MB within the zeolitic framework and its retention through non-covalent interactions [52,95]. Minor shifts in the T–O–T asymmetric stretching region (960–980 cm^−1^) suggest interactions between MB^+^ and framework Al–O^−^ sites [114,115]. Although direct identification of exchanged cations is limited by the absence of sodium detection in the XRF dataset, compositional changes observed after adsorption, including the reduction in K^+^ (0.1 ± 0.0%) and Mg^2+^ (2 ± 0.0%), the constant Ca^2+^ content (2 ± 0.0%) and the appearance of Fe^3+^ (1.42 ± 0.0%), are consistent with partial ion exchange phenomena commonly reported for cationic dye adsorption on low-silica zeolites [51,52,99,103]. Based on the combined experimental evidence from FTIR analysis, elemental composition, pH-dependent behavior, and adsorption performance trends, MB adsorption on ZEO-RS can be described as a process dominated by electrostatic attraction, pore confinement, and partial ion exchange. These mechanisms adequately explain the high adsorption efficiency and the limited reversibility observed during regeneration experiments, without invoking additional interaction pathways not directly supported by the available data [113,114,116,117].

## 3. Materials and Methods

### 3.1. Collection and Pretreatment of Residual Sands

Residual sands were collected from the coarse grit chamber of the municipal wastewater treatment plant (WWTP) of Loja, Ecuador. The material was air-dried and subsequently calcined at 500 °C for 3 h to remove organic matter. The calcined material was then ground using a vibratory disc mill and sieved to obtain a particle size below 75 µm. This fraction was used for zeolite synthesis and physicochemical characterization.

### 3.2. Zeolite Synthesis Procedure

The zeolite was synthesized using pretreated residual sands as the main Si/Al source and sodium aluminate (NaAlO_2_) as an additional aluminum precursor. Although only the final synthesis conditions are reported in this work, the development of the FAU/LTA zeolitic material involved an extensive experimental optimization process. Multiple synthesis trials were conducted by varying alkalinity, aging time, and hydrothermal treatment conditions until the desired crystalline phases and reproducible structural properties were achieved. The reported synthesis protocol corresponds to the optimized conditions that consistently yielded the target FAU-dominant dual-phase material. The synthesis mixture was formulated to maintain the following molar ratios of (SiO_2_/Al_2_O_3_) = 2, (Na_2_O/SiO_2_) = 2, and (H_2_O/Na_2_O) = 100. For every 2 g of residual sand, 3.24 g of NaOH, 81.08 g of H_2_O, and 0.81 g of NaAlO_2_ were employed. The procedure followed an adaptation of the method reported by Campoverde et al. (2023) [41]. Sodium aluminate and sodium hydroxide solutions were prepared separately and subsequently combined under continuous stirring at room temperature. The residual sand was gradually added to the alkaline mixture and stirred for 45 min. The resulting slurry was calcined at 800 °C for 5 h using a programmed temperature ramp. After cooling, the solid was rehydrated with 40 mL of deionized water and aged for 30 h at room temperature. The aged mixture was then subjected to hydrothermal treatment at 90 °C for 17 h in a sealed vessel. After synthesis, the material was vacuum-filtered and repeatedly washed with distilled water until the filtrate reached neutral pH. The zeolitic adsorbent (ZEO-RS) was synthesized in multiple independent batches following the same protocol. Prior to physicochemical characterization and adsorption/desorption experiments, the batches were homogenized to ensure material representativeness and minimize batch-to-batch variability. This procedure ensured that the reported results reflect the intrinsic behavior of the synthesized material rather than isolated synthesis events. Finally, the solid was dried at 90–105 °C for 1–2 h, labeled, and stored for further characterization.

### 3.3. Material Characterization Techniques

The chemical composition of the raw residual sand and the synthesized zeolite was determined by X-ray fluorescence (XRF) using a Bruker S1 Titan 800 handheld analyzer (Bruker, Billerica, MA, USA). Crystalline phases were identified by X-ray diffraction (XRD) using a Bruker D8 Advance A25 diffractometer (Bruker, Karlsruhe, Germany) equipped with Cu Kα radiation (λ = 0.1542 nm), operated at 40 kV and 40 mA, and scanning in the 2θ range of 4–90°. Morphology and surface texture of the materials were examined by scanning electron microscopy (SEM) using a Tescan Mira 3 field-emission microscope (Tescan, Brno, Czech Republic). Functional groups were analyzed by Fourier-transform infrared spectroscopy (FTIR) using a Jasco FT/IR-4100 spectrometer (Easton, MD, USA) in the range of 4000–550 cm^−1^. The point of zero charge (PZC) was determined using the salt addition method with NaCl solutions (0.01 and 0.05 M) over a pH range of 3–12 to evaluate the surface charge behavior of both the raw and synthesized materials. The specific surface area was measured by the nitrogen adsorption method using an automatic adsorption analyzer (Micrometrics Chemisorb 2720, Norcross, GA, USA), employing the single-point method under Ne:He gas flow ratio of 35:65%.

### 3.4. Adsorption and Desorption Experiments

The adsorption performance of methylene blue (MB) on the synthesized zeolite was evaluated under varying pH, contact time, initial dye concentration, and temperature. All experiments were conducted in batch mode using 25 mL of MB solution per test under the conditions summarized in Table 6. The selection of initial MB concentrations for the adsorption assays was based on preliminary exploratory experiments conducted prior to the systematic studies. These tests were performed to identify concentration ranges that allowed clear evaluation of the targeted adsorption phenomena without interference from early saturation or mass-transfer limitation. For kinetic and equilibrium adsorption experiments, the solution pH was adjusted and maintained at pH 7. This value was selected to represent environmentally relevant conditions commonly encountered in natural waters and treated wastewater effluents. Operating under neutral pH minimizes the need for chemical adjustment and allows a realistic assessment of adsorption performance under practical application scenarios.

All adsorption and desorption experiments were conducted in triplicate, and the reported values correspond to the arithmetic mean of three independent measurements. Experimental variability was evaluated using standard deviation, which is reported where applicable.

In the above equations q_t_, q_e_ and q_m_ (mg·g^−1^) represent the adsorption capacities at time t, at equilibrium, and the maximum adsorption capacity, respectively; c_0_, c_t_ and c_e_ (mg·L^−1^) correspond to the initial, time-dependt and equilibrium MB concentrations. The parameters k_1_ (h^−1^), k_2_ (g·mg^−1^·h^−1^) and k_t_ (mg·g^−1^·h^−1/2^) are the pseudo-first-order,pseudo-second-order and intraparticle diffusion rate constants, respectively. A (mg·g^−1^) represents the boundary-layer thickness constant. D_f_ and D_p_ denote the liquid film diffusion and particle diffusion coefficients. c_s_ (mg·L^−1^) and c_z_ (mg·kg^−1^) correspond to MB concentrations in solution and in the adsorbent. The parameter r is the average particle radius (3.7 × 10^−5^ m for < 200 mesh), and h is the film-layer thickness (1 × 10^−5^ m for a poorly stirred solution). k_L_ and k_F_ (L·mg^−1^) are the Langmuir and Freundlich constants, respectively, and r_L_ represents the favourability of the adsorption process. ΔG°, ΔH° and ΔS° (kJ·mol^−1^) correspond to Gibbs free energy, enthalpy and entropy changes derived from the van’t Hoff Equation. k_c_ is a dimensionless equilibrium constant, M_w_ is the molecular weight of adsorbate (g·mol^−1^), R is the universal gas constant (8.314 J mol^−1^·K^−1^), and T is the absolute temperature (K). 

## 4. Conclusions

This study demonstrates the successful hydrothermal transformation of wastewater treatment plant (WWTP) residual sands into a dual-phase FAU/LTA zeolitic material with well-defined structural and adsorption properties. The proposed synthesis strategy effectively valorized an abundant and underutilized waste stream, yielding a highly crystalline, low-silica aluminosilicate framework (Si/Al ≈1.7) composed predominantly of FAU domains with minor LTA contributions. This dual-phase architecture provides a high density of negatively charged sites, pronounced hydrophilicity, and significant cation-exchange capacity, which are essential attributes for the efficient removal of cationic pollutants.

Comprehensive physicochemical characterization (XRF, XRD, FTIR, SEM, and PZC) confirmed the complete structural transformation of the inert sand precursor into a stable FAU/LTA zeolitic framework. Adsorption experiments demonstrated a strong affinity of the synthesized zeolite toward methylene blue (MB), governed by a cooperative mechanism involving electrostatic attraction, hydrogen bonding, van der Waals interactions, and pore confinement, with minor contributions from chemisorptive processes such as ion exchange and Lewis acid–base interactions. Kinetic analyses revealed rapid MB uptake, initially controlled by surface interactions and subsequently limited by intraparticle diffusion within FAU supercages. Isotherm modeling using Langmuir and Freundlich equations confirmed moderately favorable and heterogeneous adsorption behavior. Thermodynamic parameters (ΔG° < 0, ΔH° = 5.26 kJ·mol^−1^, ΔS° > 0) indicated that MB adsorption is spontaneous, mildly endothermic, and predominantly entropy-driven, consistent with a physisorption-dominated process reinforced by weak chemical interactions.

Regeneration assays conducted under mild acidic, neutral and alkaline aqueous conditions revealed limited MB desorption (1–8%), indicating strong dye retention driven by steric confinement and ion-exchange stabilization within the FAU/LTA framework. Although this limits regeneration by simple pH adjustment, it ensures excellent structural stability and negligible dye leaching, which are advantageous for polishing treatments, decentralized systems, or single-use adsorption applications where environmental safety and operational simplicity are prioritized. The low regeneration efficiency does not compromise the practical relevance of the material, particularly considering its low-cost origin. Future work will explore alternative regeneration strategies, such as saline solutions or other environmentally compatible eluents, to enhance desorption while preserving framework integrity.

Overall, this work introduces a sustainable and scalable route for converting WWTP residual sands into high-performance zeolitic adsorbents. Beyond demonstrating the feasibility of using residual sands as a silicon–aluminum source, the study advances the field of waste-derived zeolites by showing that FAU/LTA dual-phase materials can deliver competitive adsorption performance, strong contaminant affinity, and remarkable structural robustness. The use of an abundant, low-cost precursor combined with a relatively simple hydrothermal synthesis process supports the potential cost-effectiveness and scalability of the proposed material. These findings highlight the promise of residual sand–based zeolites as viable candidates for sustainable wastewater purification and circular resource management applications.

## Figures and Tables

**Table 1:** Elemental composition of residual sand (RS) and synthesized zeolite (ZEO-RS) determined by XRF.

Chemical Compound	RS (%)	ZEO-RS (%)
Al_2_O_3_	13.4 ± 0.7	24.1 ± 0.6
SiO_2_	68.3 ± 0.8	41.4 ± 0.9
S	0.3 ± 0.0	<ql ^1^
K_2_O	1.0 ± 0.0	0.2 ± 0.0
MgO	<ql	2.2 ± 0.0
CaO	2.3 ± 0.0	2.2 ± 0.0
Fe_2_O_3_	1.8 ± 0.0	1.6 ± 0.0
TiO_2_	<ql	0.3 ± 0.0
MnO	<ql	0.1 ± 0.0
ZnO	<ql	0.2 ± 0.0

^1^ Below quantification limit (ql).

**Table 2:** Kinetic parameters for methylene blue adsorption onto ZEO-RS.

Model	Parameter	ZEO-RS
**Pseudo-first order**	q_e_ (mg·g^−1^)	2.04
	k_1_ (h^−1^)	0.50
	R^2^	0.85
Pseudo-second order	q_e_ (mg·g^−1^)	9.52
	k_2_ (g·mg^−1^·h^−1^)	2.73
	R^2^	0.99
Intraparticle diffusion	k_t1_ (g·mg^−1^·h^−1^)	9.37
	R^2^	0.92
	k_t2_ (g·mg^−1^·h^−1^)	0.71
	R^2^	0.95
	k_t3_ (g·mg^−1^·h^−1^)	0.05
	R^2^	0.97
Film diffusion	D_f_ (m^2^·h^−1^)	2.5 × 10^−7^
	R^2^	0.83
Particle diffusion	D_p_ (m^2^·h^−1^)	2.5 × 10^−10^
	R^2^	0.86

Experimental conditions: c_o_ MB: 20 mg·L^−1^; v: 25 mL; pH = 7; w: 0.05 g; T: 293.15 K.

**Table 3:** Langmuir and Freundlich isotherm parameters for MB adsorption onto ZEO-RS at different temperatures.

Model	Parameters	ZEO-RS		
		T = 20 °C	T = 25 °C	T = 30 °C
**Langmuir**	q_m_ (mg·g^−1^)	34.2	35.6	36.6
	k_L_ (L·g^−1^)	7.7 × 10^−3^	7.9 × 10^−3^	8.2 × 10^−3^
	r_L_	0.91–0.99	0.91–0.99	0.91–0.99
	R^2^	0.94	0.94	0.95
**Freundlich**	k_F_ (mg·g^−1^)	0.31	0.31	0.31
	n^−1^	0.87	0.87	0.8
	R^2^	0.98	0.98	0.98

**Table 4:** Thermodynamic parameters of MB adsorption on ZEO-RS.

T	ln k_c_	R^2^	ΔG°	ΔS°	ΔH°
(K)			(kJ·mol^−1^)	(kJ·mol^−1^ · K^−1^)	(kJ·mol^−1^)
293	4.91	0.99	−11.98	0.059	5.26
298	4.94		−12.25		
303	4.98		−12.56		

**Table 6:** Experimental conditions for adsorption and desorption tests.

Test	MB Content (mg·L^−1^)	pH Range	Adsorbent Dose (g)	Temperature (°C)	Contact Time	Equation
Effect of pH	5 ^a^	3–12	0.1	25 ± 1	24 h	$q_{e}=\frac{v\;x\;(c_{0}-c_{e})}{w}$ (1)
Kinetic study	20 ^b^	7	0.05	25 ± 1	15 s–24 h	Pseudo-first order $\ln\left(q_{e}-q_{t}\right)=\ln\left(q_{e}\right)-k_{1}t$ (2) Pseudo-second order $\frac{t}{q_{t}}=\frac{1}{k_{2}q_{e}^{2}}+\frac{t}{q_{e}}$ (3) Intraparticle diffusion qt=ktt12+A (4) Film diffusion $-\ln\left(1-\left(\frac{q_{t}}{q_{e}}\right)\right)=\frac{D_{f}c_{s}}{h\;r\;c_{z}}t$ (5) Particle diffusion $-\ln\left(1-{\left(\frac{q_{t}}{q_{e}}\right)}^{2}\right)=\frac{2\pi^{2}D_{p}}{r^{2}}t$ (6)
Isotherm study	1, 5, 10, 15, 20, 50, 100 and 200	7	0.1	20, 25, 30	24 h	Langmuir isotherm $\frac{c_{e}}{q_{e}}=\frac{c_{e}}{q_{m}}+\frac{1}{k_{L}q_{m}}$ (7) $r_{L}=\frac{1}{1+K_{L}c_{o}}$ (8) Freundlich isotherm $\log q_{e}=\log k_{F}+\frac{1}{n}\log c_{e}$ (9) Van’t Hoff equation $\ln k_{c}=\frac{-{∆H}^{0}}{R}\times \frac{1}{T}+\frac{{∆S}^{0}}{R}$ (10) $k_{c}=k_{L}\times M_{w}\times 1000\times 1$ (11)
Desorption study	5	3, 9, 11 ^c^	0.1	25 ± 1	24 h	

^a^ Initial concentration (5 mg·L^−1^) was selected in order to sensitively assess the influence of surface charge and solution chemistry on adsorption efficiency, while avoiding early saturation effects that could mask pH-dependent trends. ^b^ Initial concentration (20 mg·L^−1^) was deliberately chosen for the kinetic experiments to ensure a sufficient concentration driving force throughout the adsorption process. Lower concentrations led to a rapid depletion of MB at early contact times, hindering reliable monitoring of adsorption kinetics and accurate fitting of kinetic models. ^c^ Experimental approach allows the assessment of regeneration potential while preserving the structural integrity of the FAU/LTA zeolite and maintaining environmentally compatible operating conditions.

## Data Availability

The original contributions presented in this study are included in the article. Further inquiries can be directed to the corresponding author.
